# Impact of age on semen parameters in male partners of infertile couples in a rural tertiary care center of central India: A cross-sectional study

**Published:** 2017-08

**Authors:** Naina Kumar, Amit K Singh, Ajay R Choudhari

**Affiliations:** 1 *Department of Obstetrics and Gynecology, Maharishi Markandeshwar Institute of Medical Sciences and Research, Ambala, Haryana, India.*; 2 *Department of Physiology, U.P. University of Medical Sciences, Saifai, Etawah, Uttar Pradesh, India.*; 3 *Department of Physiology, Mahatma Gandhi Institute of Medical Sciences, Sewagram, Wardha, Maharashtra, India.*

**Keywords:** Fertility, Infertility, Semen analysis, Sperm, Sperm motility

## Abstract

**Background:**

High rates of sub-fertility and adverse pregnancy outcomes were seen after age 40. In contrast to oogenesis, spermatogenesis continues in elderly men.

**Objective:**

To retrospectively study the impact of aging on semen parameters in male partners of infertile couples in the rural area of developing country over 10 years and to find out whether aging affects male factor fertility and various semen parameters in this part of developing country.

**Materials and Methods:**

In this cross sectional study, the laboratory semen analysis records of 1219 male partners of infertile couples of a rural tertiary care center of Central India in a 10-year period from January 2005 to December 2014 were evaluated into 5 groups based on men age: Group 1: 21-28 yr (n=57); group 2: 29-35 yr (n=450); group 3: 36-42 yr (n=532); group 4: 43-49 yr (n=165), and group 5: 50-60 yr (n=15). Evaluation of all semen parameters were done according to WHO standard criteria (1999).

**Results:**

The analysis of semen records revealed the significant negative association of semen volume, total sperm count, sperm motility, and morphology with age. There was a significant fall in total sperm count, sperm motility, and morphology after the age of 35 yr.

**Conclusion:**

Age has significant negative effect on semen volume, total sperm count, and sperm motility and morphology in this region of India.

## Introduction

Infertility is defined by failure to achieve a clinical pregnancy after 12 months or more of regular unprotected sexual intercourse (1, 2). Males alone contribute 35-40% of infertile cases (3-5). Pathogenesis is multifactorial, any alteration to normal physiology of reproductive organs may affect sperm functions resulting in oligozoospermia (low sperm count), asthenozoospermia (loss of motility), teratozoospermia (abnormal morphology), azoospermia (sperms absence in ejaculation), oligoasthenoteratozoospermia that causes problem for successful fertilization (3, 6).

Males can contribute to conception even after 40 yr of sexual maturity (7). However, with aging degenerative changes occur in germinal epithelium, leading to fall in number, and functions of Leydig cells, thereby affecting spermatogenesis through a decrease in testosterone level (8-10). All these changes start occurring at an age of 30 yr (11). Despite these changes, the threshold age of sperm production is yet to be defined (12, 13). Semen quality has been commonly regarded as a measure of male fecundity, and changes in semen quality can occur after exposure to toxic agents or from host factor such as age (14, 15). Studies suggest that age is associated with diminished semen volume, sperm motility and/or sperm morphology, however; sperm concentration is minimally affected (16-18). 

Hence, in the present study semen parameters of male partners of infertile couples were analyzed with reference to paternal age, so as to have an idea about the effect of aging on semen parameters.

## Materials and methods

In this cross sectional study, the laboratory semen analysis records of 1219 male partners of infertile couples referred to the department of Obstetrics and Gynecology and Reproductive Biology Unit of the Physiology department of rural tertiary care center of Central India in a 10-year period from January 2005 to December 2014 were evaluated into 5 groups based on men age: Group 1: 21-28 yr (n=57); group 2: 29-35 yr (n=450); group 3: 36-42 yr (n=532); group 4: 43-49 yr (n=165), and group 5: 50-60 yr (n=15). The comparison of all semen parameters was then done following WHO standard criteria (19).

**Study subjects**

All male partners of infertile couples (infertility due to female factor/male factor/combination of two or unknown reasons) between the age group 20-60 yr were considered as study subjects who visited the gynecology department with complaints of not able to conceive since last more than one year of unprotected sexual intercourse. There were no specified inclusion or exclusion criteria, except for the age of male partner, as the data were collected retrospectively from the records. Male partners more than 60 yr were not considered for the study.

**Sample collection and semen analysis**

Semen samples were collected by masturbation into wide mouth plastic container, in a closed room in the laboratory of Reproductive Biology unit of Department of Physiology. The patients were advised to keep abstinence of around 3-4 days. Samples were then analyzed within 30-60 min, after liquefaction at 37^o^C. Semen parameters like semen volume, total sperm count, the percentage of motile and morphologically normal spermatozoa were analyzed along with the presence of pus cells in semen according to WHO criteria (19). For better confirmation of results, the findings were verified by the second observer also, before giving the final report.

**Ethical consideration**

The study was conducted after proper ethical clearance from the Institutional Ethical Committee and all attempts were made not to disclose the identity of any of the patients. As it was a retrospective study, so no written or oral consent could be obtained from the participants.

**Statistical analysis**

Statistical analysis of result was done using Statistical Package for the Social Sciences, version 20.0, SPSS Inc, Chicago, Illinois, USA (SPSS). Quantitative variables were compared using unpaired t-test/Mann-Whitney test and all the data were expressed as means±SE or percentage. Pearson’s correlation was applied to find out significant changes between age and various semen parameters and p value <0.05 was considered as significant.

## Results

The review of 1219 semen analysis of male partners of infertile couples showed different ranges of mean volume from 2.86±1.33 to 1.73±1.09, total sperm count 144.28±137.95 to 61.03±91.13 million, total progressive motility 47.47%±25.43 to 31.33%±23.87, and percentage of normal sperm cells (morphology) between 26.05%±13.26 to 19.73%±14.46. As compared to WHO criteria (19), 30.84% of samples showed below standard criteria of various semen parameters. The mean±SE values of all the semen parameters in different age groups are represented in Table I. 

**Figure 1:**
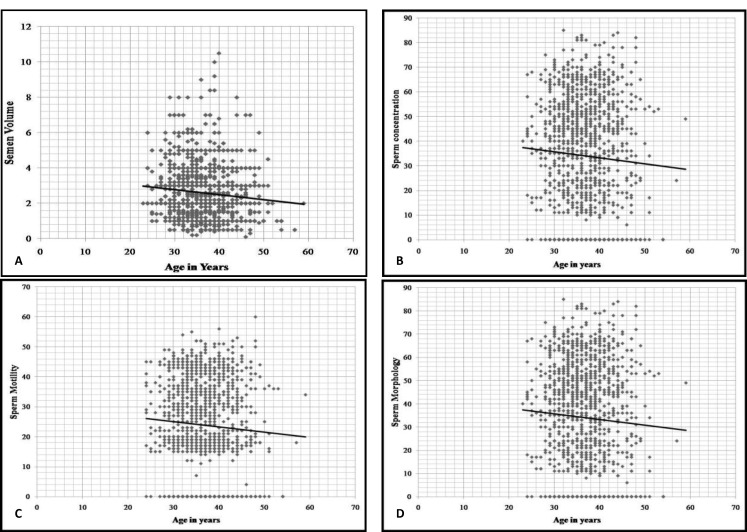
Scatter plots and linear regression lines of effect of age on semen parameters

The mean values of semen volume revealed a steady decline with increasing age. Similar negative effect of aging was noted on mean values of total sperm count, total progressive sperm motility (grade a+b), and normal sperm morphology. The maximum fall in all the semen parameters (semen volume, total sperm count, total progressive motility, and morphology) was noted after 35 yr of age as depicted in figure 1. Pearson’s correlation was applied to find out significant changes between age and semen parameters. Semen volume (r=-0.922, p=0.026), total sperm count (r=-0.93, p=0.021), motility (r=-0.95, p=0.013), and sperm morphology (r=-0.938, p=0.018) were found to be negatively correlated with increasing age, as depicted in figure 1.

**Table I:** Effect of aging on semen parameters in different age groups

**Age group (years)**	**No. of subjects (n)**	**Volume (ml)** **[Min- Max]**	**Total Sperm count (million)** **[Min-Max]**	**Motility (Grade a+b) (%)** **[Min-Max]**	**Morphology (%)** **[Min-Max]**
21-28	57	2.86±1.33 [1-6]	144.28±137.95 [0.2-414]	47.47±25.43 [0-88]	26.05±13.26 [0-49]
29-35	450	2.74±1.48 [0.2-8]	149.46±173.72 [0.1-1358]	48.14±30.39 [0-95]	24.8±15.66 [0-55]
36-42	532	2.48±1.50 [0.4-10.5]	120.41±156.68 [0.2-949]	40.0±27.14 [0-83]	23.07±15.93 [0-56]
43-49	165	2.44±1.37 [0.1-8]	112.33±142.74 [0.2-776]	33.12±25.06 [0-84]	22.99±16.47 [0-60]
50-60	15	1.73±1.09 [0.5-4.5]	61.03±91.13 [0.1-328.5]	31.33±23.87 [0-59]	19.73±14.46 [0-37]

**A: **Scatter plots and linear regression line showing declining trend of semen volume with age (p=0.026)

**B: **Scatter plots and linear regression line showing declining trend of total sperm concentration with age (p=0.021)

**C:** Scatter plots and linear regression line showing declining trend of sperm motility with age (p=0.013)

**D: **Scatter plots and linear regression lines showing declining trend of sperm morphology with age (p=0.018)

## Discussion

The present study clearly reveals that age has a significant negative effect on semen volume, total sperm count, sperm motility, and morphology. It is well known that maternal aging contributes significantly to human infertility, whereas males continue to produce sperms even at advanced age, allowing them to reproduce during senescence (20, 21). Although very little is known, paternal age may also contribute to human infertility. In 1969, Sasano and Ichijo first described the decrease in sperm concentration as men age and found that 90% of seminiferous tubules in men between 20-30 yr of age contained spermatids, whereas 50% of seminiferous tubules of men between 40-50 yr had spermatids (22). Only 10% of seminiferous tubules from men aged >80 years contained spermatids (22). 

Similar to our study results, a literature review on the association between male age and semen quality which compared 30-year-old men to 50-year-old men found decrease in semen volume (3-22%), sperm motility (3-37%), and percent of normal sperm (4-18%), but on contrary to our findings, they found that the age has no effect on sperm concentration (23). Similar results were also observed in another study that evaluated men between 22-80 yr of age (18). Many recent studies also reveal that sperm quality in men decreases with age (21, 24-28). In addition, advanced paternal age has been implicated in an increased frequency of miscarriages, autosomal dominant disorders, aneuploidies, and other diseases (24, 29-34).

Further in a study of 8515 planned pregnancies (of greater than 24 wk gestation), it was observed that men older than 35 yr had half the chance of fathering a child within 12 months compared with men aged less than 25 yr (35). Moreover various retrospective studies in the USA, China and India showed age related effects in semen parameters following the WHO guideline (23, 27, 36). In general, retrospective data implies declined sperm counts through ages (37, 38).

Our study revealed a declining trend in total sperm counts with aging, this is similar to many studies which indicate fall in all the semen parameters with advancing male age (18, 23, 39). There are many studies that also reveal either increase or even decrease in sperm concentration with aging. For example, a study reported a decrease in sperm concentration of up to 3.3% per year of age (40). Another study including 1283 men, who cryo-banked sperm prior to vasectomy, sperm concentrations were found to be lower at both extremes of age as compared to men aged 26-45 yr (41). Contrary to this, a study comprising of 22,759 infertile men in North-eastern Spain revealed a statistically significant increase in the sperm concentration of 0.7% per year of age. This amounts to an increase in the concentration of 14% over a 20-year period (42). 

In contrast to concentration, several study results support the finding that sperm motility decreases with advancing age. Our study also observed a significant fall in sperm motility with aging. Several other studies reported a statistically significant reduction in motility by 0.17-0.6% per year with aging resulting in 3-12% fall in motility over 20 yr (40, 41). Moreover, a recent study revealed that sperm motility decreases by 0.8% per year of age and linear motion decreases by 0.2% per year (43).

In addition to motility, morphology also appears to be affected with increasing male age. The present study also revealed a decline in sperm morphology with age. Similar results were found in studies that indicate a decline in normal sperm morphology of 0.2-0.9% per year of age, resulting in a 4-18% decrease in normal morphology over a 20-year period (40, 42). This was further supported by studies conducted in various parts of India that revealed diminished sperm quality with age (36, 44). 

Furthermore, our study showed fall in semen volume with age, which is supported by evidence which suggests that there is a mild decrease in seminal volume with increasing age, although the clinical significance of this finding is marginal (45). The decrease in volume may be related to seminal vesicle insufficiency because seminal vesicle fluid composes most of the ejaculate volume (23, 46). Most of the data suggested that the pronounced changes occur in men aged >45 yr. Semen volume declines from a mean of 2.80 ml in those aged 45-47.8 yr to 1.95 ml in men more than 56.6 yr (18, 47). Table II depicts various study results revealing the effect of aging on semen parameters which support our study results.

Hence, understanding the impact of male age on fertility has become increasingly salient in public health as growing number of men are choosing to have children at an older age (17) and many couples need an assisted reproduction procedure to have a child (55, 56).

**Table II:** Various studies depicting effect of age on semen parameters

**Studys**	**No. of subjects**	**Significant findings**
Levitas *et al* 2007 (48)	6022	Excellent semen quality at the age of 30-35 years, Sharp reduction in all parameters after 55 years
Cardona Maya *et al* 2009 (49)	1364	The decrease in semen volume, motility, sperm concentration, with increasing age
Harris *et al* 2011 (45)	Review	Age related decline in fertility rate and an increase in DNA breakdown in sperms
Silva *et al* 2012 (50)	975	Age related changes in nuclear vacuoles and DNA damage
Stone *et al* 2013 (51)	4822	Total sperm count and normal morphology declined after 40 years, Sperm motility and semen volume decline after 43 and 45 years of age
Purandhar *et al* 2013 (52)	90	Fall in enzymatic antioxidants and neutrophil count with increasing age. The decline in motility, vitality, and morphology with aging
Omran *et al* 2013 (53)	52	Fall in sperm density, motility, normal morphology, total antioxidant capacity and DNA integrity with aging
Priyadarsini *et al *2014 (54)	733	Fall in progressive motility, vitality, and morphology with aging. No significant changes were found in semen volume and total count

**Limitation**

The limitation of this study is that it is a retrospective analysis of data. Moreover, we took only age into consideration, but there can be many other factors like occupation, temperature, drugs, etc. that can affect the semen parameters and overall male fertility. So, in future keeping this as base information, we can conduct prospective analysis considering other factors also that can affect male fertility. Hence, we can work in that direction so as to prevent such factors that can seriously affect the male fertility.

## Conclusion

Hence, aging has a significant negative impact on various semen parameters and male fertility in this region of India. The older men in infertile couples have lower semen parameters resulting in lower fertility potential.
